# Tailoring the Microstructure of Sol–Gel Derived Hydroxyapatite/Zirconia Nanocrystalline Composites

**DOI:** 10.1007/s11671-010-9766-z

**Published:** 2010-08-31

**Authors:** HC Vasconcelos, MC Barreto

**Affiliations:** 1 Department of Technological Sciences and Development, Campus de Ponta Delgada, Azores University, 9501-801, Ponta Delgada, Açores, Portugal; 2 Physics Department of FCT/UNL, CEFITEC—Centre of Physics and Technological Research, Quinta da Torre, 2829-516, Caparica, Portugal; 3 CIRN—Centre of Research in Natural Resources, 9501-801, Ponta Delgada, Açores, Portugal

**Keywords:** Hydroxyapatite, Zirconia, Sol–gel, Tailored microstructure, Water vapor atmosphere

## Abstract

In this study, we tailor the microstructure of hydroxyapatite/zirconia nanocrystalline composites by optimizing processing parameters, namely, introducing an atmosphere of water vapor during sintering in order to control the thermal stability of hydroxyapatite, and a modified sol–gel process that yields to an excellent intergranular distribution of zirconia phase dispersed intergranularly within the hydroxyapatite matrix. In terms of mechanical behavior, SEM images of fissure deflection and the presence of monoclinic ZrO_2_ content on cracked surface indicate that both toughening mechanisms, stress-induced tetragonal to monoclinic phase transformation and deflection, are active for toughness enhancement.

## Introduction

Hydroxyapatite [HA, Ca_10_(PO_4_)_6_(OH)_2_] has attracted much interest as a biomaterial for use in prosthetic applications due to the similarity of its crystallography and chemical composition to that of human hard tissues [1,2]. However, the main weakness of this material lies in its poor mechanical strength that makes it unsuitable for load-bearing applications.

An attractive way of producing tougher HA is to use composites of 3 mol% yttria-stabilized tetragonal zirconia (YSZ) and HA, where the biocompatibility and bioactivity come from the apatite phase and the high strength is derived from the zirconia oxide (ZrO_2_) phase [3-5], on account of its high strength and fracture toughness being significantly increased by stress-induced tetragonal (t) to monoclinic (m) phase transformation toughening, or by deflection toughening mechanism [6-10].

Therefore, such composites must display uniform microstructures with a high degree of dispersion and without decomposition of the HA, during the sintering process. However, it has been reported that the addition of ZrO_2_ causes an increase in the content of tricalcium phosphate (β-TCP, Ca_3_(PO_4_)_2_) [11], which seriously deteriorates the mechanical properties and chemical stability of these composites. In addition, calcium can be released from HA and interact with zirconium, resulting in the formation of cubic zirconia or calcium zirconate, which inhibits the toughening transformation mechanism [4,12-15].

To minimize these reactions, some efforts have been made toward reducing the sintering temperature and holding time. One alternative is the use of HA and YSZ nanopowders [16]. Another important method is the introduction of external pressure using the following techniques: spark plasma sintering (SPS), hot pressing or hot isostatic pressing (HIP) [5,16-18]. However, the exact mechanism of β-TCP content increase in ZrO_2_-added HA is not fully understood, and thus a method to control this decomposition has not been reported so far.

Sol–gel technique has been selected as a potential method to synthesize a large variety of materials [19-22], and in particularly ceramic matrix composites carefully doped with additional phases, offering a very good homogeneity and a better control of the morphology and microstructure. Several synthesis routes have been proposed for the synthesis of HA as well as different mixing conditions with numerous reactants molar ratios [23-26]. Although various precursors have been tried in the attempt to obtain a well-developed HA, the Ca(NO_3_)_2_ and [P(OC_2_H_5_)_3_] (TEP) combination has shown the most promising results, but until now it has still been difficult to obtain phase-pure HA.

In this study, a new type of HA/YSZ composite was fabricated with tailored microstructure by optimizing processing parameters in order to control the thermal stability of HA and YSZ grain phase distribution. An important outcome of the present work is an excellent intergranular distribution of YSZ phase, for the first time, in ZrO_2_-based composites.

## Experimental

High-purity chemicals of calcium nitrate tetrahydrate (Ca(NO_3_)_2_4H_2_O), triethyl phosphate (P(OC_2_H_5_)_3_) bought from Sigma–Aldrich, ammonium hydroxide (NH_4_OH, 30% BDH, England), yttria-stabilized (3wt% Y_2_O_3_) tetragonal zirconia (TZ-3Y, Tosoh Corporation) with particle size as 40 nm and BET as 16 ± 3 m^2^/g were used as starting ingredients for HA/YSZ composites.

**Figure 1:**
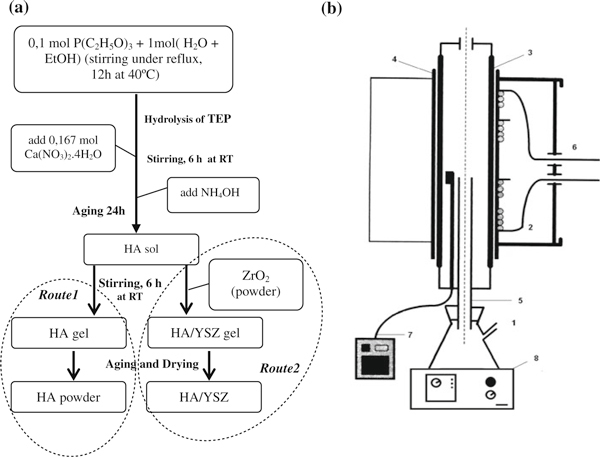
**(a) Flow chart for the production of HA (*route1*) plus HA with zirconia powders (HA/YSZ) (*route2*) and (b) set-up of the water vapor atmosphere-assisted sintering arrangement**: *1* Erlenmeyer with distilled water (T ~ 100°C), *2* Electric furnace, *3* Al_2_O_3_ tube (large), *4* SiO_2_-Al_2_O_3_ tube, *5* Al_2_O_3_ tube (small), *6* Kenthal resistance, *7* thermocouple, *8* Hot plate.

Gel powders of HA and HA/YSZ composites (containing 10 mol% YSZ, denoted by HAZ10) were processed by a tailored sol–gel processing route, according the scheme illustrate in Figure 1a, involving triethyl phosphate (TEP) and calcium nitrate (Ca(NO_3_)_2_) as precursors of HA (*route1*). In order to obtain HAZ10 (*route2*), YSZ commercial powder diluted in deionised water was added to the HA sol.

At the end of both routes, the solvents were then driven off at 60°C until a viscous liquid was obtained. Further drying of the viscous liquid at 60°C resulted in a gel. Oven drying was undertaken for a further 24 h at 120°C, and powders (pure HA and HAZ10, respectively) were obtained. Green pellets were prepared by uniaxial pressing at 100 MPa, and sintering was performed in water vapor atmosphere at the temperature of 950°C for 1 h. Under typical atmospheric conditions, water vapor was continuously generated by the evaporation of boiling liquid water and directly introduced inside the furnace, using the set-up illustrated in Figure 1b.

The characterization of as-sintered samples was carried out by scanning electron microscopy (SEM/EDX, JEOL JMS-840) and X-ray diffraction (XRD) using CuKα radiation (Siemens D-5000 diffractometer). Data were collected from 25 to 63° 2θ with step of 0.02° and counting time 12 s. The identification of phases present was done using JCPDS files n° 9-432 to HA phase, 17-923 to ZrO_2_ tetragonal phase and 13-307 to ZrO_2_ monoclinic phase.

Vickers indentations (using a Shimadzu Micro Hardness Tester Type-M) and resulting crack propagation were used to detect toughening mechanisms.

## Results and Discussion

**Figure 2:**
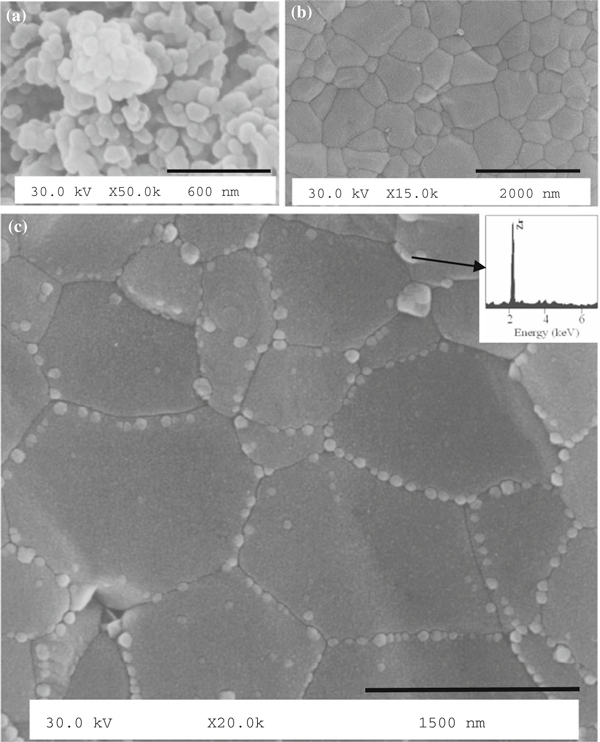
**SEM micrographs of (a) nanocrystalline HA powders prepared by sol–gel (after *route1*), (b) polished cross-section of HA water vapor-assisted sintered compact (950°C for 1 h) showing a dense and uniform microstructure, (c) polished cross-section of HA/YSZ water vapor-assisted sintered compacts (950°C for 1 h), showing a tailored microstructure**. The *inset* shows the EDX spectra acquired from the individual zirconia grains.

SEM on the HA powder, after drying for 24 h at 120°C, is shown in Figure 2a. The averaged particle size was ~60–100 nm, and the particles were agglomerated.

Figure 2b, c illustrates the scanning electron microstructure of the polished and thermally etched surface of the sintered HA and HAZ10, respectively. Full densification of the optimized powders and respective compacts (load = 100 MPa), after *routes 1* and *2*, was achieved in 1 h at low temperature of 950°C. In Figure 2c, the dark grains are HA and the bright ones are ZrO_2_, which were dispersed mostly intergranularly within the HA matrix. EDX analysis (inset of Figure 2c) indicates the presence of Zr within whitish grain. Due to process synthesis, zirconia served as nucleation sites during HA precipitation, so HA crystals were formed on the surfaces of ZrO_2_ particles. This phenomenon provided a more intimate mixing in binary composites, yielding a higher dispersion, allowing ZrO_2_ particles to be present mostly at grain boundaries, without agglomerates. Thus, the prepared samples were fully densified with small isolated voids, as shown in Figure 2c.

It is well established that HA is thermally decomposed into mostly β-TCP [Ca_3_(PO_4_)_2_], CaO and water vapor [27-29], according to the following reactions:

(1) $${\text{Ca}}_{10}{\left({\text{PO}}_{4}\right)}_{6}{\left(\text{OH}\right)}_{2}\Leftrightarrow {\text{Ca}}_{10}{\left({\text{PO}}_{4}\right)}_{6}{\left(\text{OH}\right)}_{\left(2-2x\right)}{\text{O}}_{x}+↑{\text{H}}_{2}\text{O}$$

(2) $${\text{Ca}}_{10}{\left({\text{PO}}_{4}\right)}_{6}\text{O}\Leftrightarrow 2\beta -{\text{Ca}}_{3}{\left({\text{PO}}_{4}\right)}_{2}+{\text{Ca}}_{4}{\text{P}}_{2}{\text{O}}_{9}$$

(3) $${\text{Ca}}_{10}{\left({\text{PO}}_{4}\right)}_{6}{(\text{OH)}}_{2}\Leftrightarrow 3\beta -{\text{Ca}}_{3}{\left({\text{PO}}_{4}\right)}_{2}+\text{CaO}\;+↑{\text{H}}_{2}\text{O}$$

Also, the tetragonal phase of ZrO_2_ can be decomposed through the reaction [28]:

(4) $${\text{Ca}}_{10}{\left({\text{PO}}_{4}\right)}_{6}{(\text{OH)}}_{\left(2-2x\right)}{\text{O}}_{x}+y{\text{ZrO}}_{2(\text{tetragonal})}\Leftrightarrow 3{\text{Ca}}_{3}{\left({\text{PO}}_{4}\right)}_{2}+\text{CaO}{\left({\text{ZrO}}_{2(\text{cubic})}\right)}_{y}+↑{\text{H}}_{2}\text{O}$$

**Figure 3:**
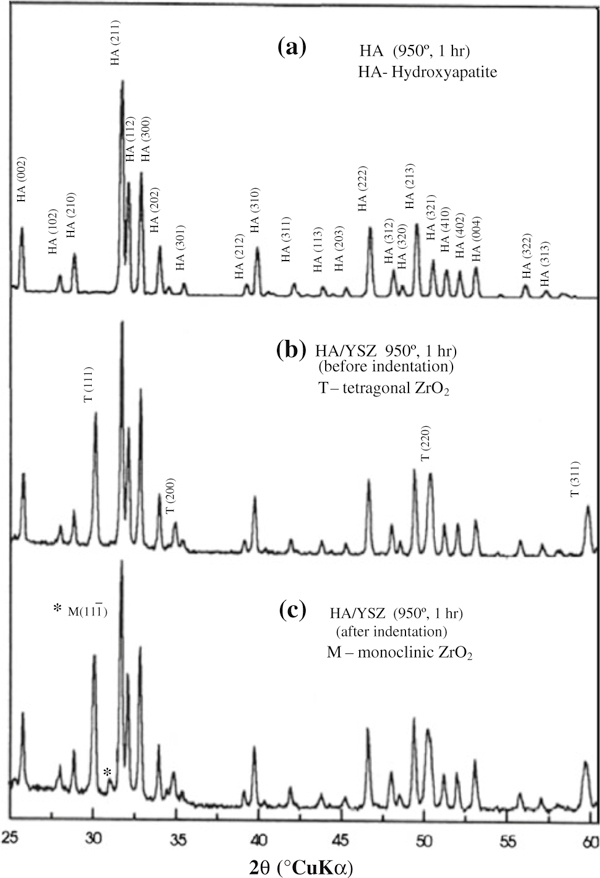
**(a) XRD patterns of HA and YSZ/HA powder, with Ca/P ratio of 1,67 and YSZ content of 10%, heated at 950°C, 1 h in water vapor atmosphere, before indentation (b) and after indentation (c)**.

However, the obtained HA and HAZ10 compacts did not contain any phases other than HA and the tetragonal modification of zirconia, as revealed by their X-ray powder diffraction patterns in Figure 3a, b. The patterns demonstrate the stable nature of HA; peaks indicating the presence of stoichiometric HA after sinterization at 950°C for 1 h.

Two mechanisms may explain this behavior: first, since a gaseous species (H_2_O) exists on the products side of the decomposition reactions, sintering atmosphere would be expected to influence the decomposition kinetics of HA. In the present work, a water vapor atmosphere was used during sintering reaction, causing a compensation of the partial vapor atmosphere of water inside the furnace, avoiding vacancies formation in the HA structure—Ca_10_(PO_4_)_6_(OH)_(2–2*x*)_O_*x*_ at reaction (1), counteracting the effect of HA decomposition in the reactions (2), (3) and (4), and secondly the decomposition reactions of HA were avoided, most probably because diffusion of water from the reaction zone to the surfaces is retarded by the zirconia matrix (nano intergranular ZrO_2_ particles) in boundaries of HA grains, forming a continuous framework.

**Figure 4:**
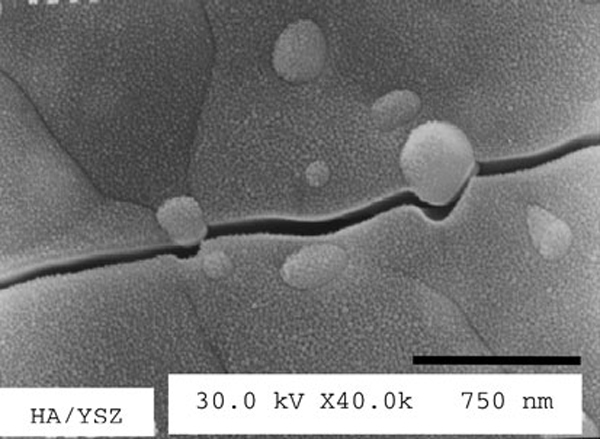
**Indentation crack propagation, during indentation fracture, revealing active crack deflection by the reinforcing phase**.

The mechanical properties of this category of composite materials can be optimized by carefully tailoring the microstructure. Thus, the contribution of stress-induced phase transformation was evaluated by XRD, which is assessed in terms of the reflection of ZrO_2_ monoclinic phase, as shown in Figure 3c. The presence of m-ZrO_2_ content on cracked surface (after Vickers indentation) indicates that the transformation toughening phenomenon is an active mechanism for toughness enhancement. Additionally, crack deflection toughening by ZrO_2_ particles also contributes to toughening of the composite. SEM observations also sustain the role of crack deflection toughening in these composites. The observation of indentation crack in the HAZ10 composite (Figure 4) shows little crack deflection around the dispersed ZrO_2_ particles; therefore, this mechanism seems also contribute to the toughening.

## Conclusions

In this study, HA was reinforced with 3 mol% of Y_2_O_3_ partially stabilized ZrO_2,_ and structure-tailored HAZ10 composites, yielding intergranular distribution of ZrO_2_ particles, were fabricated by a modified sol–gel process. Absence of deleterious reaction products is mostly attributed to the sintering atmosphere of water vapor and tailored microstructure. Stress-induced ZrO_2_ phase transformation (indicated by XRD) together with SEM images of fissure deflection indicates that both mechanisms are active for toughness enhancement.
